# Effect of cotrimoxazole prophylaxis on the incidence of malaria in HIV-infected children in 2012, in Abidjan, Côte d’Ivoire: a prospective cohort study

**DOI:** 10.1186/s12879-015-1009-6

**Published:** 2015-08-07

**Authors:** Aïda Mounkaila Harouna, Madeleine Amorissani-Folquet, François Tanoh Eboua, Sophie Desmonde, Sylvie N’Gbeche, Edmond Addi Aka, Kouakou Kouadio, Brou Kouacou, Karen Malateste, Clarisse Bosse-Amani, Patrick Ahuatchi Coffie, Valeriane Leroy, for the IeDEA paediatric West African Study Group

**Affiliations:** Inserm U897 – Epidémiologie – Biostatistiques, F-33000 Bordeaux, France; University Bordeaux, ISPED, Centre Inserm, U897 – Epidémiologie – Biostatistiques, F-33000 Bordeaux, France; University Félix Houphouët Boigny, Abidjan, Côte d’Ivoire; Paediatrics, Félix Houphouët Boigny University Hospital, Abidjan, Côte d’Ivoire; Paediatrics, Yopougon University Hospital, Abidjan, Côte d’Ivoire; CePReF-enfant, ACONDA, Abidjan, Côte d’Ivoire; CIRBA, Abidjan, Côte d’Ivoire; Centre MTCT-plus, Abidjan, Côte d’Ivoire; Département de Dermatologie et d’Infectiologie, Université Félix Houphouët Boigny, Abidjan, Côte d’Ivoire

**Keywords:** Malaria, HIV, Cotrimoxazole, Antiretroviral therapy, Children, Africa

## Abstract

**Background:**

Cotrimoxazole prophylaxis has an antimalarial effect which could have an additional protective effect against malaria in HIV-infected children on antiretroviral therapy (ART). We measured the incidence and associated factors of malaria in HIV-infected children on ART and/or cotrimoxazole in Abidjan, Côte d’Ivoire.

**Methods:**

All HIV-infected children <16 years, followed-up in the IeDEA West-African paediatric cohort (pWADA) in Abidjan, were prospectively included from May to August 2012, the rainy season. Children presenting signs suggesting malaria had a thick blood smear and were classified as confirmed or probable malaria. We calculated incidence density rates (IR) per 100 child-years (CY). Risk factors were assessed using a Poisson regression model.

**Results:**

Overall, 1117 children were included, of whom 89 % were ART-treated and 67 % received cotrimoxazole. Overall, there were 51 malaria events occurring in 48 children: 28 confirmed and 23 probable; 94 % were uncomplicated malaria. The overall IR of malaria (confirmed and probable) was 18.3/100 CY (95 % CI: 13.3-23.4), varying from 4.2/100 CY (95 % CI: 1.1-7.3) in children on ART and cotrimoxazole to 57.3/100 CY (95 % CI: 7.1-107.6) for those receiving no treatment at all. In univariate analysis, age <5 years was significantly associated with a 2-fold IR of malaria compared to age >10 years (incidence rate ratio [IRR] = 2.18, 95 % CI: 1.04-4.58). Adjusted for severe immunodeficiency, cotrimoxazole reduced significantly the IR of first malarial episode (adjusted IRR [aIRR] = 0.13, 95 % CI: 0.02-0.69 and aIRR = 0.05, 95 % CI:0.02-0.18 in those off and on ART respectively). Severe immunodeficiency increased significantly the malaria IR (aIRR = 4.03, 95 % CI: 1.55-10.47).

When considering the IR of confirmed malaria only, this varied from 2.4/100 CY (95 % CI: 0.0-4.8) in children on ART and cotrimoxazole to 34.4/100 CY (95 % CI: 0.0-73.3) for those receiving no treatment at all. In adjusted analyses, the IR of malaria in children on both cotrimoxazole and ART was significantly reduced (aIRR = 0.05, 95 % CI: 0.01-0.24) compared to those receiving no treatment at all.

**Conclusions:**

Cotrimoxazole prophylaxis was strongly protective against the incidence of malaria when associated with ART in HIV-infected children. Thus, these drugs should be provided as widely and durably as possible in all HIV-infected children <5 years of age.

## Background

In 2010, approximately 216 million malaria episodes occurred worldwide, 81 % of which were in sub-Saharan Africa. Overall, 655,000 of these cases led to death, 86 % occurring in children under five years [1]. That same year, there were approximately 3.4 million children under fifteen years living with HIV worldwide of which more than 90 % lived in sub-Saharan Africa [2]. Malaria and HIV are two infections with overlapping epidemiologic maps that to be addressed urgently.

There is an interaction between malaria and HIV infections which causes a global health problem [3]. On the one hand, HIV-related immunodeficiency results in children being more vulnerable to malaria, reducing the efficacy of antimalarial drugs [4, 5]. On the other hand, malaria is known to increase viral replication and thus HIV disease progression [3]. This interaction needs to be further investigated in a context where access to cotrimoxazole prophylaxis and antiretroviral therapy (ART) could have a protective effect against malaria in HIV infected-children [6, 7]. Although not defined as an opportunistic disease [8, 4], a malarial episode may become more serious with HIV influence as reported by many studies mostly conducted in Southern and Eastern Africa [5, 7, 9, 10]. Before the ART era, cotrimoxazole prophylaxis reduced morbidity and mortality in children with HIV by preventing bacterial infections, diarrhea, malaria, and Pneumocystis jirovecii pneumonia, in Zambia [11].

In Côte d’Ivoire, malaria is the first cause of morbidity and mortality in children: the incidence was 288 ‰ in 2012 [12] in children under five years and represented in 2010, 25 % of death causes [13]. Moreover, in 2009, HIV represented 4.4 % of deaths in children under five in Côte d’Ivoire [2]. Despite this, the incidence of malaria in HIV-infected children, treated or not, has never been documented in West Africa, where the malaria endemic, microbial resistance and HIV prevalence are different compared to other regions. The main objective of this study was to measure the incidence of malaria and associated factors in HIV-infected children on ART and/or cotrimoxazole prophylaxis and who are followed-up in HIV-care programmes in Abidjan, Côte d’Ivoire.

## Methods

### Study sites

The paediatric West African Database on AIDS (pWADA), a component of the International epidemiologic Databases to Evaluate AIDS (IeDEA), is a multi-cohort collaboration including 11 paediatric HIV-care programmes in seven West African countries, including five centres in Abidjan. The main objective of this cohort collaboration is to document the long-term morbidity, mortality and retention of HIV-infected children under 16 years [14]. In each centre, HIV-infected children are seen in the context of a routine visit at least once every three months and/or because they present symptoms requiring clinical care. CD4 counts are measured every six months. Viral load is not routinely monitored. ART, cotrimoxazole prophylaxis and HIV-related blood analyses are free of charge. However, other health care benefits are partially or fully paid for by patient.

The present study, called PALUVIH (Malaria and HIV study), was conducted within the five centres of pWADA cohort in Abidjan: two in Yopougon (the Centre pédiatrique de Prise en charge, de Recherche et de Formation [CePReF-enfant] and the paediatric cohort of the Yopougon University Hospital), one in Treichville (the Centre Intégré de Recherche Bioclinique d’Abidjan [CIRBA]), one in Cocody (the paediatric cohort of the Cocody University Hospital) and one in Abobo (the Mother To Child Transmission centre [MTCT-Plus]). In 2012, the incidence of malaria in Abidjan varied in the general population, ranging from 215 ‰ in Cocody area to 112 ‰ for Yopougon; in Treichville it was 118 ‰ and 185 ‰ in Abobo. Thus, Cocody and Abobo districts were a 2-fold higher endemic areas for malaria compared to other districts in Abidjan [12].

### Study design and patient population

We conducted a prospective cohort study, nested in the Abidjan pWADA cohort, from May to August 2012 during the first rainy season of the year, favourable to the reproduction of mosquitoes (malaria vectors). In Abidjan, there are two rainy seasons: May-July and October-November.

All HIV-infected children (aged <16 years) followed-up within pWADA and who attended, for any reason, at least once one of the five centres during the study period were included in the PALUVIH cohort from 7th May 2012 (the date of the routine implementation of malaria documentation) and were followed-up until 6th August 2012. In the pWADA cohort, children with any inter-current clinical symptoms between two per-protocol visits, which are at three-month intervals, can attend their centre.

During the PALUVIH study period, we prospectively documented all clinical episodes at any per-protocol or inter current visits evoking malaria in children included (fever, chills, aches, headache, diarrhoea, vomiting, irritability, confusion…), with a thick blood smear, the first-line exam recommended by WHO and the Ivoirian National Program to diagnose malaria [15].

### Definition

The outcome was the incidence of incident malaria, defined according to WHO, as follows [16, 17]:

According to the embodiment of thick blood smear: confirmed malaria (clinical symptoms suggesting malaria documented by a positive thick blood smear) and probable malaria (clinical events without laboratory confirmation or negative thick blood smear but solved by an antimalarial treatment).

According to the presence or not of clinical or biological severity signs of malaria: uncomplicated malaria (no clinical or biological signs of severity) and severe malaria (one or more of the following clinical or biological signs of severity: consciousness disorders (Blantyre coma scale ≤3), prostration, inability to eat, >2 convulsions/24 h, respiratory distress, cardiovascular collapse, clinical jaundice accompanied by other signs of vital organ dysfunction, oliguria <0.5 mL/kg/h, serum créatinine >265 μmol/L, haemoglobinuria, spontaneous bleeding disorders, pulmonary oedema, hypoglycemia (<2.2 mmol/L), metabolic acidosis (bicarbonate <15 mmol/L); severe anaemia (Hb <5 g/dL); hyperparasitemia (>5 %) and hyperlactacidemia (lactic acid >5 mmol/L)) [15].

A recurrent event of malaria was defined as any malarial event occurring in a child who had already presented malaria during the study period and for whom the event had been resolved, confirmed by a negative control.

Thick blood smears and all complementary tests (parasitemia, biochemistry and hematology tests) and antimalarial treatments were paid by the PALUVIH project and there were no extra costs for patient families. Cases of uncomplicated malaria were treated per os by Artesunate-Amodiaquine or Artemether-Lumefantrine association and complicated cases with injectable Artemether or Quinine Resorcine.

### Ethical approval

The IeDEA West Africa collaboration was approved by the national ethics committees of each participating country and the name of the Ivoirian ethics committee where this study took place is as follows : “Comité National d’Ethique et de la Recherche de République de Côte d’Ivoire”. As care was conducted according to the national standard of care recommended, individual informed signed consent was waived for this sub-study.

### Data collection

Data were collected retrospectively and prospectively using two collection instruments:

Socio-demographic and HIV-infection data for all children at the time of the PALUVIH study inclusion were extracted from the pWADA databases (birth’s date; HIV type; last CD4 cell count, or percentage available and date);

A register was used to collect prospectively during the study period for all children, date and reason of consultation, ongoing cotrimoxazole and ART regimen;

A standardized form was used prospectively for all children presenting any sign suggesting malaria to collect the following data: clinical symptoms; temperature; insecticide-treated bed-nets use; taking medication before consultation; paraclinical examination results (thick blood smear and parasitemia, full blood count,…); existence of clinical or biological severity signs; existence of any comorbidity; malaria episode management (hospitalization, outpatient treatment); evolution of the malaria episode (control thick blood smear performed 14 days after antimalarial treatment start, healing, short-term sequelae, death).

### Data management and statistical analysis

Categorical variables were presented as frequencies and continuous variables as median with Interquartile Range (IQR).

Incidence density rates (IR) of all malaria events (confirmed and probable, then only confirmed to provide conservative estimates), including both first and recurrent events, were calculated per 100 child-years of follow-up (CY) with their 95 % Confidence Interval (95 % CI) according to the Miettinen formula [18]. Each child included contributed to the denominator for the three-month study period (from May to August 2012) or until the death if this occurred earlier. The baseline characteristics were defined at time of inclusion (7th May 2012). Factors associated with the incidence of first case of malaria were identified by a Poisson regression model including the following co-variables: cotrimoxazole, ART, age group (<5 years, [5-10[years and ≥10 years) and immunodeficiency for age at the time of visit (+/− 3 months) (severe, moderate and no immunodeficiency). We defined severe immunodeficiency for age according to the 2010 WHO recommendations for ART initiation [19] as follows: CD4 < 25 % if aged < 1 year; CD4 < 20 % if aged between [1–3 years[; CD4 < 15 % if aged between [3-5years[; CD4 < 200/mm^3^ if aged ≥5 years. Moderate immunodeficiency was defined as follow: CD4 [25 %-35 % [if aged <1 year; CD4 [20 %-30 %[if aged [1–3 years[; CD4 [15 %-25 %[if aged between [3-5years[; CD4 between [200/mm^3^-500/mm^3^ [if aged ≥5 years. This regression analysis was first conducted with all malaria cases, and then only confirmed malaria cases in a sensitivity analysis.

Because children could present more than one malaria episode during the study period, we limited this specific analysis to the incidence of the first episode. We first ran univariate analyses for each co-variable, and then ran a full multivariate model estimating adjusted incidence rate ratio (aIRR). Due to the small number of events, only major explanatory variables were kept in multivariate analyses allowing the model to converge. Thus, the adjusted model included only the following covariates: ART (forced as the main explanatory variable), cotrimoxazole and severe immunodeficiency for age. Data were entered using Microsoft Access 2003 and statistical analyses were performed using Epiinfo version 3.5.3 and SAS version 9.3.

## Results

### Baseline characteristics

Overall, 1117 children were included. Children aged <5 years accounted for 13 % of the population and had a median CD4 % of 29 % (IQR: 24–35) while older children aged ≥5 years had 781 CD4 cells/mm^3^ (IQR: 507–1084). In the MTCT-Plus centre, 28 % of the included children were under five compared to the other centres where this proportion was on average 12 % (*p* < 0.01). Cotrimoxazole prophylaxis was prescribed to 67 % of the children and ART to 89 % among whom 84 % received a Non-Nucleoside Reverse Transcriptase Inhibitor (NNRTI)-based regimen and 14 % a Protease-Inhibitor (PI)-based regimen (Table 1).

**Table 1:** Baseline characteristics of the 1117 HIV-infected children included in the pWADA cohort, Abidjan, May-August 2012

Characteristics (N = 1117)	n (%)
**Centre participants, n (%)**	
Cocody University Hospital	182 (16.3)
Yopougon University Hospital	344 (30.8)
CePReF	322 (28.8)
CIRBA	212 (19.0)
MTCT+	57 (5.1)
Total site	1117 (100.0)
**Age (years)**	
Median (IQR)	9 (6–12)
**Sex, n (%)**	
Male	553 (49.5)
Female	564 (50.5)
**HIV type, n (%)**	
1	1111 (99.4)
2	4 (0.4)
1 & 2	2 (0.2)
**Last CD4 measurement available at the time of the visit**	
Percentage when age < 5 years n (%)	144 (13.0)
Median CD4 % (IQR)	29 (24–35)
CD4 > 35 %, n (%)	34 (23.6)
25 % ≤ CD4 ≤ 35 %, n (%)	66 (45.8)
CD4 < 25 %, n (%)	40 (27.8)
Missing, n (%)	4 (2.8)
Cell count when age ≥ 5 years n (%)	973 (87.0)
Median CD4 cells/mm^3^, (IQR)	781 (507–1084)
CD4 > 500 cells/mm^3^, n (%)	731 (75.1)
350 ≤ CD4 ≤ 500 cells/mm^3^, n (%)	102 (10.5)
CD4 < 350 cells/mm^3^, n (%)	140 (14.4)
Missing, n (%)	0 (0.0)
**Treatment received at the time of the visit**	
On ART – On Cotrimoxazole	667 (59.7)
Off ART – On Cotrimoxazole	83 (7.4)
On ART – Off Cotrimoxazole	323 (29.0)
Off ART – Off Cotrimoxazole	35 (3.1)
On ART unknown for Cotrimoxazole	9 (0.8)
**ART drugs regimen**	
2 NRTI^a^ + 1 NNRTI^b^	842 (84.3)
2 NRTI + 1 PI^c^	144 (14.4)
3 NRTI	9 (0.9)
Missing	4 (0.4)
Median follow-up time on ART (year) (IQR)	2.2 (0.4-5.2)
Median follow-up time on Cotrimoxazole (year) (IQR)	3.4 (1.3-6.1)

n: number; ART: antiretroviral therapy; NRTI^a^: Nucleoside Reverse Transcriptase Inhibitors. INNRT^b^: Non-Nucleoside Reverse Transcriptase Inhibitors. PI^c^: Protease Inhibitors

### Malaria cases description and evolution

During the study, 48 children presented 51 malaria attacks; three children had a recurrent episode. Six children (13 %) used an insecticide-treated bed-net at the time of the event; but this information was not available for the whole sample.

Seven children (15 %) were administered by their caregivers a known antimalarial drug before consultation, of whom six had taken Artemeter-Luméfantrine or Artesunate-Amodiaquine.

Among the 51 cases of malaria, 28 were confirmed by a thick blood smear and the median parasitemia was 1800/μL (IQR: 440–4210); the 23 remaining cases were classified as probable malaria. Complete blood counts were performed for 30 children, among which median haemoglobin (Hb) was 10.2 g/dL (IQR: 8.8-11.3) and 80 % had haemoglobin <12 g/dL.

Overall, 48 of the 51 diagnoses were uncomplicated malaria, among them, eight were treated in outpatient day-care hospitals and for the remaining 40, stander outpatient treatment was the modality of care. The three cases of severe malaria were treated with hospitalization. Among these severe malarial events, two were associated with severe anemia (Hb <7 g/dl) and one with a cardiovascular collapse. The overall distribution of antimalarial treatment prescriptions were as follows: Arthemether-Lumefrantrine per os (42 %); Artesunate-Amodiaquine per os (35 %); Arthemether injections (13 %); Quinine injections (10 %). In eleven cases, unknown antibiotics were prescribed in addition to the antimalarial treatment. None of the malarial events led to death. Of the 28 cases of confirmed malaria, 75 % of thick blood smears were controlled two weeks after the beginning of the antimalarial treatment, of which two came back positive.

### Incidence density rates of malaria

The overall IR of malaria (confirmed and probable) was estimated to be 18.3/100 CY (95 % IC: 13.3-23.4). IRs increased from 4.2/100 CY (95 % CI: 1.1-7.3) in children on ART and cotrimoxazole to 57.3/100 CY (95 % CI: 7.1-107.6) in those without ART or cotrimoxazole. It was 14.5/100 CY (95 % CI: 0.0-30.9) in children on cotrimoxazole.

The IR in children with severe immunodeficiency for age was 29.4/100 CY (95 % CI: 3.6-55.2) and 17.0/100 CY (95 % CI: 11.7-22.3) in those without severe immunodeficiency.

Among children under five, the IR was 30.7/100 CY (95 % CI: 12.5-48.8) and 18.1/100 CY (95 % CI: 10.2-26.1) in children aged between [5–10 years].

Compared to the other sites, MTCT-Plus had the highest observed IR of 77.5/100 CY (95 % CI: 31.7-123.2) (Table 2).

**Table 2:** Incidence density rates of malaria per 100 child-years of follow-up in the 1117 HIV-infected children included in the pWADA cohort, Abidjan, May-August 2012

Characteristics	N	Number of child-years of follow-up	Incidence density rate with 95 % Confidence Interval for all cases of malaria: probable, confirmed including recurrences			Incidence density rate with 95 % Confidence Interval for confirmed malaria including recurrences		
			n (%)	Incidence density rate (per 100 child-years)	(95 % Confidence Interval)	n’ (%)	Incidence density rate (per 100 child-years)	(95 % Confidence Interval)
**Incidence density rate of malaria**								
Cases without recurrences	1117	278	48 (94.1)	17.2	(12.4-22.1)	-	-	-
Recurrence cases	1117	278	3 (5.9)	1.1	(0.0-2.3)	-	-	-
Confirmed	1117	278	28 (55.0)	10.1	(6.3-13.8)	-	-	-
Probable	1117	278	23 (45.0)	8.3	(4.9-11.6)	-	-	-
Overall (confirmed and probable)			51 (100.0)	18.3	(13.3-23.4)	-	-	-
	1117	278						
**Incidence density rate of malaria according to treatment**								
On ART – On Cotrimoxazole	667	166	7 (13.7)	4.2	(1.1-7.3)	4 (14.3)	2.4	(0.0-4.8)
Off ART – On Cotrimoxazole	83	21	3 (5.9)	14.5	(0.0-30.9)	2 (7.1)	9.7	(0.0-23.1)
On ART – Off Cotrimoxazole	323	80	36 (70.6)	44.7	(30.1-59.3)	19 (67.9)	23.6	(13.0-34.2)
Off ART – Off Cotrimoxazole	35	9	5 (9.8)	57.3	(7.1-107.6)	3 (10.7)	34.4	(0.0-73.3)
**Immunodeficiency according to age** ^a^								
Severe	69	17	5 (9.8)	29.4	(3.61-55.2)	3 (10.7)	17.5	(0.0-37.2)
Moderate	106	26	6 (11.8)	23.1	(4.6-41.5)	4 (14.3)	15.1	(0.3-30.0)
No/Missing	942	235	40 (78.4)	17.0	(11.7-22.3)	21 (75.0)	8.9	(5.1-12.8)
**Age**								
<5 years	144	36	11 (21.6)	30.7	(12.5-48.8)	6 (21.4)	16.7	(3.3-30.1)
[5-10[years	443	110	20 (39.2)	18.1	(10.2-26.1)	12 (42.9)	10.9	(4.7-17.0)
≥10 years	530	132	20 (39.2)	15.1	(8.5-21.8)	10 (35.7)	7.6	(2.9-12.3)
**Centres**								
University hospital of Cocody	182	45	1 (2.0)	2.2	(0.0-6.5)	1 (3.6)	2.2	(0.0-6.5)
University hospital of Yopougon	344	86	9 (17.6)	10.5	(3.6-17.4)	4 (14.3)	4.7	(0.1-9.2)
CePReF	322	80	23 (45.1)	28.7	(17.0-40.4)	11 (39.3)	13.7	(5.6-21.8)
CIRBA	212	53	7 (13.7)	13.3	(3.4-23.1)	2 (7.1)	3.8	(0.0-9.0)
MTCT-Plus	57	14	11 (21.6)	77.5	(31.7-123.2)	10 (35.7)	70.4	(26.8-114.1)

^a^Using te 2010 WHO definition. ART: antiretroviral therapy; n’() number and percentage of only confirmed malaria cases

When considering only confirmed malaria cases, IR were lower (Table 2).

### Factors associated with the incidence of the first malaria event

When analysing the 48 cases of first malaria episode (confirmed and probable), in univariate analyses, compared to children receiving no treatment at all, the incidence of malaria was respectively reduced by 93 % among children receiving both cotrimoxazole prophylaxis and ART (incidence rate ratio [IRR]: 0.07, 95 % CI: 0.02-0.21) and by 84 % in those on cotrimoxazole alone (IRR: 0.16, 95 % CI: 0.03-0.80), (Table 3).

**Table 3:** Factors associated with the incidence of the first malaria event (n = 48) in the 1117 HIV-infected children included in the pWADA cohort. Abidjan May-August 2012

	N	n	Univariate analysis			Adjusted analysis		
			IRR	(95 % IC)	*p*-value	aIRR	(95 % CI)	*p*-value
**Antiretroviral therapy (ART) and cotrimoxazole exposure**					<0.01			<0.01
On ART - On cotrimoxazole	667	7	0.07	(0.02-0.21)	<0.01	0.05	(0.02-0.18)	<0.01
Off ART - On cotrimoxazole	83	2	0.16	(0.03-0.80)	0.03	0.13	(0.02-0.69)	0.02
On ART - Off cotrimoxazole	332	34	0.69	(0.27-1.77)	0.44	0.69	(0.27-1.77)	0.44
Off ART - Off cotrimoxazole	35	5	1	-	-	1	-	-
**Immunodeficiency for Age** ^a^					0.38			0.02
Severe	69	5	1.87	(0.73-4.76)	0.19	4.03	(1.55-10.47)	<0.01
Moderate	106	6	1.44	(0.61-3.40)	0.41	2.14	(0.89-5.14)	0.09
No/Missing	942	40	1	-	-	1	-	-
**Age**					0.14			
<5 years	144	11	2.18	(1.04-4.58)	0.04		Not included	
[5-10[years	443	18	1.13	(0.59-2.16)	0.70			
≥10 years	530	19	1	-	-			
**Centres**					<0.01			
CHU de Cocody	182	1	1	-	-			
CHU de Yopougon	344	9	4.80	(0.61-37.87)	0.14		Not included	
CePReF	322	20	11.66	(1.56-86.91)	0.02			
CIRBA	212	7	6.11	(0.75-49.65)	0.09			
MTCT-Plus	57	11	38.72	(5.00-299.97)	<0.01			

N: number of children; n: number of malaria incident cases (confirmed and probable); IRR: incidence rate ratio; aIRR, adjusted incidence rate ratio; 95 % CI:95 % Confidence Interval. ^a^Using the 2010 WHO definition

There was a non-significant trend to a higher malaria incidence in severely immunodeficient children compared to those not immunodeficient (IRR: 1.87, 95 % CI: 0.73-4.76).

Age < 5 years was also significantly associated with a higher incidence of malaria (IRR: 2.18, 95 % CI: 1.04-4.58) compared to children aged > 10 years. Incidence of malaria was also significantly associated with the follow-up clinic (*p* < 0.01): the MTCT-Plus care centre where the children were also the youngest with a median age of two years, was significantly associated with a higher incidence of malaria (IRR: 38.72, 95 % CI: 5.00-299.97).

Unfortunately, due to the low number of malaria events, our model did not converge in multivariate analyses, when including in the final model all of the above variables associated in univariate analysis. Thus, we privileged the analysis of the effect of ART and cotrimoxazole adjusted on the severe immunodeficiency to further explore our question while the effect of age is already well known.

In the multivariate analysis adjusted for both severe immunodeficiency and ART, cotrimoxazole prophylaxis either alone or in combination with ART reduced significantly the risk of malaria by 87 %, (adjusted incidence rate ratio [aIRR]: 0.13, 95 % CI: (0.02-0.69), and 95 % (aIRR: 0.05, 95 % CI: 0.02-0.18), respectively) (Table 3).

ART alone tended to reduce the incidence of malaria by 31 % (aIRR: 0.69 (95 % CI: 0.27-1.77) but this reduction was not statistically significant. The adjusted incidence of malaria was significantly highest in children with severe immunodeficiency for age when adjusted for ART and cotrimoxazole prophylaxis (aIRR = 4.03, 95 % CI: 1.55-10.47) (Table 3).

When conducting the analysis only including the confirmed cases, malaria incidence was no longer reduced in children on cotrimoxazole alone in the univariate analysis (IRR: 0.13, 95 % CI: 0.01-1.25) (Table 4). We observed similar results in the multivariate analyses, adjusted for immunodeficiency, where cotrimoxazole prophylaxis in combination with ART still reduced significantly the risk of malaria by 95 %, (adjusted incidence rate ratio [aIRR]: 0.05, 95 % CI: (0.01-0.24), but the use of cotrimoxazole alone was not longer significantly associated with malaria (aIRR: 0.11 (95 % CI: 0.01-1.08) (Table 4).

**Table 4:** Factors associated with the incidence of the first confirmed malaria event (n = 25) in the 1117 HIV-infected children included in the pWADA cohort. Abidjan May-August 2012

	N	n	Univariate analysis			Adjusted analysis		
			IRR	(95 % IC)	*p*-value	aIRR	(95 % CI)	*p*-value
**Antiretroviral therapy (ART) and cotrimoxazole exposure**					<0.01			<0.01
On ART - On cotrimoxazole	667	4	0.06	(0.01-0.28)	<0.01	0.05	(0.01-0.24)	<0.01
Off ART - On cotrimoxazole	83	1	0.13	(0.01-1.25)	0.08	0.11	(0.01-1.08)	0.06
On ART - Off cotrimoxazole	332	17	0.58	(0.17-1.99)	0.38	0.58	(0.17-1.99)	0.39
Off ART - Off cotrimoxazole	35	3	1	-	-	1	-	-
**Immunodeficiency for Age** ^a^					0.48			0.09
Severe	69	3	2.19	(0.65-7.39)	0.21	4.59	(1.32-15.98)	0.02
Moderate	106	3	1.40	(0.41-4.73)	0.59	2.09	(0.61-7.19)	0.24
No/Missing	942	19	1	-	-	1	-	-
**Age**					0.17			
<5 years	144	6	2.82	(0.98-8.13)	0.05		Not included	
[5-10[years	443	11	1.64	(0.66-4.09)	0.28			
≥10 years	530	8	1	-	-			
**Centres**					<0.01			
CHU de Cocody	182	1	1	-	-			
CHU de Yopougon	344	4	2.13	(0.24-19.08)	0.50		Not included	
CePReF	322	8	4.66	(0.58-37.30)	0.15			
CIRBA	212	2	1.74	(1.56-19.25)	0.65			
MTCT-Plus	57	10	35.20	(4.51-275.03)	<0.01			

N: number of children; n: number of malaria incident cases (only confirmed); IRR: incidence rate ratio; aIRR, adjusted incidence rate ratio; 95 % CI:95 % Confidence Interval. ^a^Using the 2010 WHO definition

## Discussion

To our knowledge, this study is the first to specifically describe the association between the intake of cotrimoxazole prophylaxis and the incidence of malaria in HIV-infected children in several care programs in Abidjan. It has allowed documenting in a standardized manner any suspected malaria cases among HIV-infected children. We reported a high incidence rate of malaria (18.3/100 CY) that was strongly reduced in children on cotrimoxazole alone or combined with ART when adjusting for severe immunodeficiency. Incidence of malaria was twice as high in children under five years compared to those older than 10 years. The incidence of malaria was not reduced in children treated with ART only compared to those not on ART.

Our study was conducted in a substantial sample size of more than 1000 HIV-infected children during the rainy season, a high transmission period of malaria.

However, we also note study limitations. First, our estimation of the incidence of malaria is likely to have been underestimated for several reasons: due to logistical reasons, our study period did not cover the entire rainy season, which usually ends in November. We only documented all clinically driven symptoms but did not performed routine thick smears in asymptomatic children. We may also have missed malaria cases because they could have been treated outside the study centres or because of self-medication for malaria before seeking care, which is often practiced in Côte d’Ivoire, as reported in 15 % of the cases in our study. This could have led to false negative results of the thick blood smear performed also contributing to underestimation of the incidence of malaria. Second, in this study we used the gold standard for malaria diagnosis, which is microscopy. In a high endemic setting, where the population is immunocompromised, the use of additional laboratory tests such as rapid diagnostic testing or PCR could have allowed identifying additional cases of malaria, but this was not feasible for logistical reasons. Finally, there may also be a classification bias over-estimating the incidence of malaria: we defined probable malaria as any clinical event evoking malaria and solved by antimalarial, however 23 % were co-treated with antibiotics which could indicate that the event may have been another infection, solved by the antibiotics the child was co-treated with. Nevertheless, we conducted this study over a short period of time with a previous awareness campaign among the medical staff to improve and standardise all the diagnosis procedures of malaria. Consequently we conclude that our estimation is quite a reasonable minimal estimate. Unfortunately, also for logistical reasons, it was no feasible to compare these results with a group of HIV-uninfected children in the same community.

In our study, the global incidence of malaria (confirmed and probable) in HIV-1-infected children was 183 ‰ and 307 ‰ in children under five, respectively. This estimation seems to be higher than others reported in Cote d’Ivoire. The country is ranked the fifth among countries most affected by malaria compared to the rest of the world with an incidence estimated to be 116 ‰ in the general population in 2012, with a similar risk in children less than five: 288 ‰ [12]. In this same context, a previous study conducted in HIV-infected children before ART initiation estimated a morbidity incidence of 66 ‰ attributable to malaria cases based on a syndromic approach [20]. Our study shows a significant association between malaria incidence and cotrimoxazole in HIV-infected children with a strong independent protective effect reducing by up to 87 % the risk of malaria as shown by previous studies [21–26]. We also report that the risk for malaria was significantly lower in those treated with both ART and cotrimoxazole, reduced by up to 95 % compared to those not treated. Mermin *et al.* has also reported this previously in an adult population in Uganda [27]. If used alone, ART tended to reduce the incidence of malaria by 31 % in our study although this was not statistically significant. Several other studies, conducted *in vitro*, have shown a significantly protective effect of protease inhibitor drugs [28–31]. This difference could be explained by two factors: (i) a probable lack of statistical power in our study; (ii) only 14 % of our children were treated with a PI-based regimen known to have a direct antimalarial activity [32].

Severe immunodeficiency is also known as a risk factor for malaria [9, 33, 34]. Consistently, our results show that children with severe immunodeficiency have a 4-fold higher adjusted risk to develop malaria than those not severely immunodeficient.

Children <5 years have a twice higher risk to develop malaria in our study and this is expected and highly consistent with what was reported by the WHO [1].

Our results suggest that cotrimoxazole provides an ongoing beneficial protection against malaria after immune reconstitution in ART-treated children. This justifies its use for all children even if they are receiving ART, with a beneficial additive effect, as recommended by WHO since 2000 [35].

Likewise, we reported the frequency of malaria occurrence in children already severely immunodeficient, suggesting that the use of ART must be widespread to all HIV-infected children with the aim to restore immunity as recommended by WHO in 2013 [36]. In addition, children under five are most at risk for malaria, extra attention should be given to this target population by increasing, for example, the use of insecticide-treated bed-nets in addition to treatments. This latter measure remains an essential measure to implement, as we reported that only 12 % of children presenting malaria in our study used it in practice. This use should significantly be improved to reduce malaria-related morbidity and mortality in Africa [37].

## Conclusion

In this study, we measured incidence density rates of malaria in HIV-infected children and analysed its associated factors. Cotrimoxazole prophylaxis strongly protected against malaria. We did not show a significant association between incidence of malaria and ART. But cotrimoxazole prophylaxis protection is more important if used in addition to ART. Also, immunodeficiency was an important risk factor of malaria incidence suggesting that the indirect effect of ART in restoring immunity justifies its use for all HIV-infected children. These two drugs should be provided as widely and as durably as possible in all children, as recommended by WHO [36]. Likewise, the provision of insecticide-treated bed-nets could reduce the incidence of malaria even further in children under five, which are more affected by malaria. These interventions will be helpful in reaching the sixth millennium development goals to improve child health [38].

## Abbreviations

aIRR: adjusted Incidence Rate Ratio
IR: Incidence density Rate
ART: Antiretroviral therapy
CePReF-enfant: Centre de Prise en charge de Recherche et de Formation, volet enfant
CI: Confidence interval
CIRBA: Centre Intégré de Recherches Biocliniques d’Abidjan
CY: Child-Years
Hb: Haemoglobin
HIV: Human Immunodeficiency Virus
IeDEA: International epidemiologic Databases to Evaluate AIDS
Inserm: Institut national de la santé et de la recherche médicale
IP: Protease-Inhibitor based regimen
IQR: InterQuartile Range
IR: Incidence density Rate
IRR: Incidence Rate Ratio
MTCT: Mother To Child Transmission
NNRTI: Non-Nucleoside Reverse Transcriptase Inhibitor based regimen
PALUVIH: Malaria on HIV field study
pWADA: paediatric West African Database on AIDS
Univ.: University
